# RenderGAN: Generating Realistic Labeled Data

**DOI:** 10.3389/frobt.2018.00066

**Published:** 2018-06-08

**Authors:** Leon Sixt, Benjamin Wild, Tim Landgraf

**Affiliations:** Fachbereich Mathematik und Informatik, Freie Universität Berlin, Berlin, Germany

**Keywords:** generative adversarial networks, unsupervised learning, social insects, markers, deep learning

## Abstract

Deep Convolutional Neuronal Networks (DCNNs) are showing remarkable performance on many computer vision tasks. Due to their large parameter space, they require many labeled samples when trained in a supervised setting. The costs of annotating data manually can render the use of DCNNs infeasible. We present a novel framework called RenderGAN that can generate large amounts of realistic, labeled images by combining a 3D model and the Generative Adversarial Network framework. In our approach, image augmentations (e.g., lighting, background, and detail) are learned from unlabeled data such that the generated images are strikingly realistic while preserving the labels known from the 3D model. We apply the RenderGAN framework to generate images of barcode-like markers that are attached to honeybees. Training a DCNN on data generated by the RenderGAN yields considerably better performance than training it on various baselines.

## 1. Introduction

When an image is taken from a real-world scene, many factors determine the final appearance: background, lighting, object shape, position, and orientation of the object, the noise of the camera sensor, and more. In computer vision, high-level information such as class, shape, or pose is reconstructed from raw image data. Most real-world applications require the reconstruction to be invariant to noise, background, and lighting changes.

In recent years, deep convolutional neural networks (DCNNs) advanced to the state of the art in many computer vision tasks (Krizhevsky et al., 2012; Razavian et al., 2014; He et al., 2015). More training data usually increases the performance of DCNNs. While image data is mostly abundant, labels for supervised training must often be created manually—a time-consuming and tedious activity. For complex annotations such as human joint angles, camera viewpoint, or image segmentation, the costs of labeling can be prohibitive.

In our project BeesBook (Wario et al., 2015), we extract and analyze the social network of a honeybee colony. The bees are monitored with multiple cameras and can be identified by a unique binary marker attached on their back. To track the bees, it is required to decode the binary marker reliably. The recent successes of DCNNs (Russakovsky et al., 2015) implied that a DCNN could decode bees markers if enough training samples are available. However, annotating an image of a single marker takes about a minute. If we labeled only 10 images for each of the 4,096 different IDs, we would have to work more than 680 h, i.e., several months of tedious labor. It would still be questionable if 10 samples could reliably represent the image variance due to different lighting conditions and object rotations in space. Thus, the main question is *how can we acquire more labeled data*?

A simple solution could have been to annotate more data by crowdsourcing the task. This would likely have created several months of work for distributing the data, and collecting and processing the labels. Another intuitive approach would be to train a DCNN on images generated by a 3D renderer. We used a simple 3D model (no lighting, shading) but could not train a DCNN which performed well on real image data.

Here we propose a solution based on a modification to the recently proposed GAN framework (Goodfellow et al., 2014). We use a simple 3D model of the binary marker to generate an idealistic image of the marker. The generator network then learns to add the missing image detail such as lighting, background, and image noise so that the resulting images look strikingly real. By ensuring that the generator cannot change the ID label, we can collect realistic images from the generator with their corresponding labels we fed to the 3D model. This dataset can then be used to train a DCNN which performs well on real data.

In the next section, we shortly review the state-of-the-art in generating realistic images. Subsequently, we describe our RenderGAN method and compare the results of a DCNN trained to decode the binary marker with different baselines. Finally, we discuss our results in the BeesBook project, and in a wider context of generating realistic data with preserved labels.

## 2. Related work

A common approach to deal with limited amount of labels is data augmentation (Goodfellow et al., 2016, Chapter 7.4). Translation, noise, and other deformations can often be applied without changing the labels, thereby effectively increasing the number of training samples and reducing overfitting. Ratner et al. (2017) propose to automatically learn augmentations with GANs.

DCNNs learn a hierarchy of features—many of which are applicable to related domains (Yosinski et al., 2014). Therefore, a common technique is to pre-train a model on a larger dataset such as ImageNet (Deng et al., 2009) and then fine-tune its parameters to the task at hand (Girshick et al., 2014; Razavian et al., 2014; Long et al., 2015). This technique only works in cases where a large enough related dataset exists. Furthermore, labeling enough data for fine-tuning might still be costly.

If a basic model of the data exists (for example, a 3D model of the human body), it can be used to generate labeled data. Peng et al. (2015) generated training data for a DCNN with 3D-CAD models. Su et al. (2015) used 3D-CAD models from large online repositories to generate large amounts of training images for the viewpoint estimation task on the PASCAL 3D+ dataset (Xiang et al., 2014). Zhou et al. (2016) also use the PASCAL 3D+ dataset to learn the dense flow prediction between images. Wu et al. (2016) construct a network to learn a 3D skeleton of objects such as chairs. Massa et al. (2015) are matching natural images to 3D-CAD models with features extracted from a DCNN. Richter et al. (2016) and Ros et al. (2016) used 3D game engines to collect labeled data for image segmentation. However, the explicit modeling of the image acquisition physics (scene lighting, reflections, lense distortions, sensor noise, etc.) is cumbersome and might still not be able to fully reproduce the particularities of the imaging process such as unstructured background or object specific noise. Training a DCNN on generated data that misses certain features will result in overfitting and poor performance on the real data.

**Figure 1:**
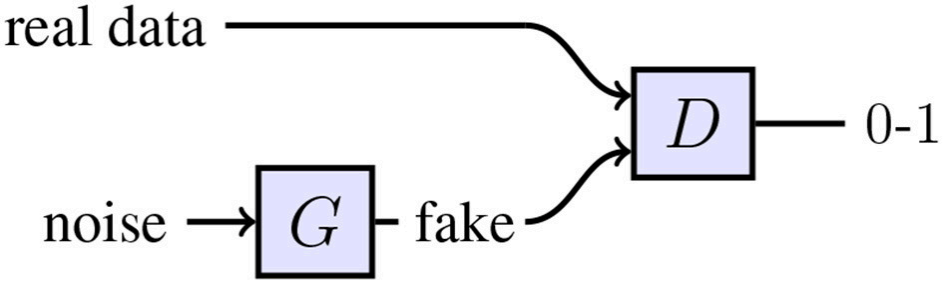
Topology of a GAN. The discriminator network *D* is trained to distinguish between “fake” and real data. The generator network *G* receives a random vector as input. *G* is optimized to maximize the chance of the discriminator making a mistake.

Generative Adversarial Networks (GAN) (see Figure 1) can learn to generate high-quality samples (Goodfellow et al., 2014), i.e., sample from the data distribution *p*(*x*). Denton et al. (2015) synthesized images with a GAN on the CIFAR dataset (Krizhevsky, 2009), which were hard for humans to distinguish from real images. While a GAN implicitly learns a meaningful latent embedding of the data (Radford et al., 2015), there is no simple relationship between the latent dimensions and the labels of interest. Therefore, high-level information can't be inferred from generated samples. cGANs are an extension of GANs to sample from a conditional distribution given some labels, i.e., *p*(*x*|*l*). However, training cGANs requires a labeled dataset. Springenberg (2015) showed that GANs can be used in a semi-supervised setting but restricted their analysis to categorical labels. Wang and Gupta (2016) trained two separate GANs, one to model the object normals and another one for the texture conditioned on the normals. As they rely on conditional GANs, they need large amounts of labeled data. Chen et al. (2016) used an information theoretic to disentangle the representation. They decomposed the representation into a structured and unstructured part. And successfully related on a qualitative level the structured part to high-level concepts such as camera viewpoint or hair style. However, explicitly controlling the relationship between the latent space and generated samples *without using labeled data* is an open problem, i.e., sampling from *p*(*x, l*) without requiring labels for training.

Independent work by Shrivastava et al. (2016) proposes to postprocess images of a 3D model of eyes and hand poses with a GAN. In contrast to our work, they propose a L1-loss to ensure that the labels remain valid.

Planar markers with binary codes have been shown to be feasible for tracking large groups of insects. A system, previously developed for ants (Mersch et al., 2013) was shown to successfully track 100 bees for 2 days Blut et al. (2017). The markers in used were originally described as fiducial markers in augmented reality systems (Fiala, 2005) and rely on spatial derivatives to detect the rectangular outline of a tag. A similar system using flat and rectangular markers for tracking larger insects was also proposed and might be adapted to honeybees (Crall et al., 2015). This system binarizes the image globally and searches for rectangular regions representing the corners of the marker. The previous BeesBook vision system (Wario et al., 2015) was tailored to specifically track all animals of small honeybee colonies over their entire lifetime. It uses a round and curved marker and searches for ellipse-shaped edge formations.

## 3. RenderGAN

In the BeesBook project (Wario et al., 2015), we need to decode the position, orientation and binary id of markers attached to honeybees in a hive. Due to the large amount of honeybees this task can't be done manually. Although we invested a substantial amount of time on labeling, a DCNN trained on the limited labeled dataset did not perform well. We therefore wanted to synthesize labeled images which are realistic enough to train an improved decoder network.

### 3.1. 3D model

**Figure 2:**
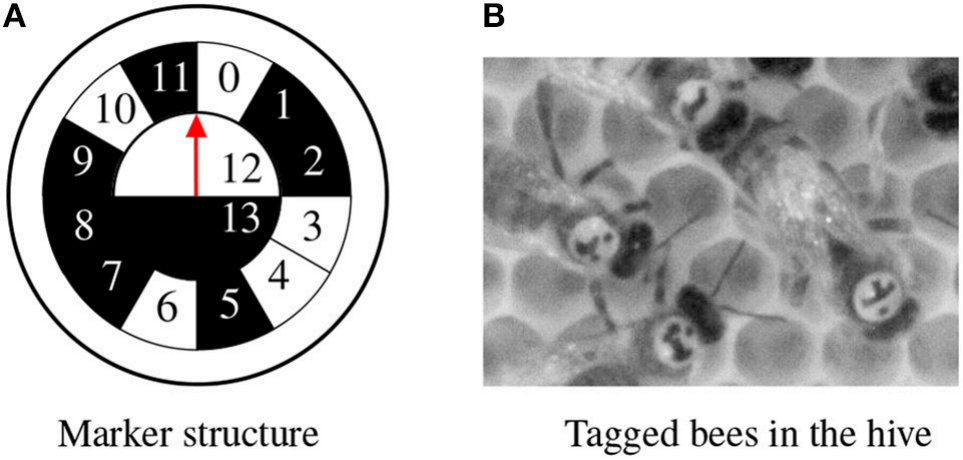
**(A)** The marker represents a unique binary code (cell 0–11) and encodes the orientation with the semicircles 12 and 13. The red arrow points toward the head of the bee. This marker encodes the id 100110100010. **(B)** Cutout from a high-resolution image.

**Figure 3:**
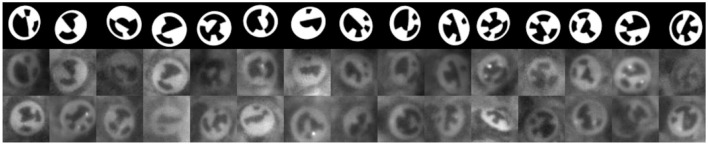
**First row:** Images from the 3D model without augmentation. **Below:** Corresponding images from the RenderGAN. **Last row:** Real images of bee's markers.

We use a 3D Model to generate an idealistic image of the marker. The 3D model comprises a mesh which represents the structure of the marker and a simple camera model to project the mesh to the image plane (see Figure 2). The model is parameterized by its position, pitch, yaw and roll, and bit assignment. Given a parameter set, the 3D model is rendered to an image of the marker, a background segmentation mask and a depth map. The generated images is an idealistic representation of the marker and lacks many important factors: blur, lighting, background, and image detail (see Figure 3). A DCNN trained on data from this idealistic model did not generalize to real images.

The discriminator gradients cannot be backpropagated through the 3D model. Still, we want to learn the distributions of the inputs of the 3D model such as the orientation and position. While there exists differentiable renders (Loper and Black, 2014), we found it the easiest to train a neural network to emulate the 3D model. Its outputs are indistinguishable from the images of the 3D model. Yet, the neural network allows backpropagation of the gradients. The weights of the 3D model network are fixed during the GAN training. The bit assignments are sampled uniformly.

### 3.2. Augmentations

Normally augmentations are used to enlarge and diversify the training data. Typical augmentations are translation, rotation, adding noise, and scaling of pixel intensities. The parameters of the augmentations are normally sampled from a random distribution. Here, one has to ensure that the augmentations don't change the content of the image. For example, adding too much noise can occlude the object.

In the RenderGAN, we would like to learn the parameters to a set of augmentation such that a simple 3D model is morphed to a realistic image. We have to ensure that all augmentations preserve the high-level information.

**Figure 4:**
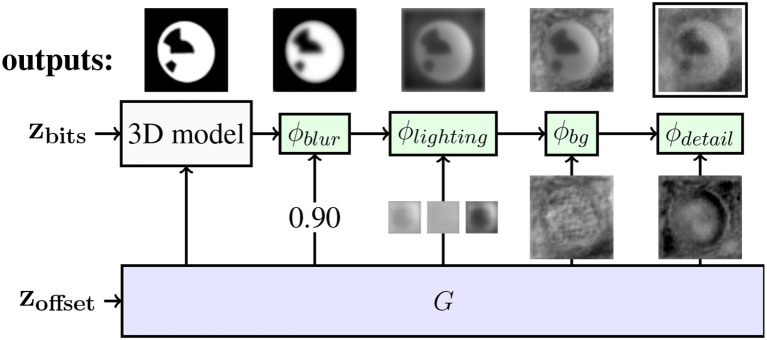
Augmentations of the RenderGAN applied to the BeesBook project. The arrows from *G* to the augmentations ϕ depict the inputs to the augmentations. The generator provides the position and orientations to the 3D model, whereas the bits are sampled uniformly. On top, the output of each stage is shown. The output of ϕ_*detail*_ is forwarded to the discriminator.

We apply different augmentations that account for blur, lighting, background, and image detail. The output of the 3D model and of each augmentation is of the same shape as the image. In Figure 4, the structure of the generator is shown.

#### 3.2.1. Blurriness

The 3D model produces hard edges, but the images of the real tags show a broad range of blur. The generator produces a scalar α ∈ [0, 1] per image that controls the blur.

(1) $$\begin{array}{r} \phi_{blur}\left(x,\alpha \right)=\left(1-\alpha \right)\left(x-b_{\sigma }\left(x\right)\right)+b_{\sigma }\left(x\right) \end{array}$$

where *b*_σ_(*x*) = *x* ∗ *k*_σ_ denotes convolving the image *x* with a Gaussian kernel *k*_σ_ of scale σ. The implementation of the blur function is inspired by Laplacian pyramids (Burt and Adelson, 1983). As required for augmentations, the labels are preserved, because we limit the maximum amount of blur by picking σ = 2. ϕ_*blur*_ is also differentiable w.r.t the inputs α and *x*.

#### 3.2.2. Lighting of the tag

The images from the 3D model are binary. In real images, tags exhibit different shades of gray. We model the lighting by a smooth scaling and shifting of the pixel intensities. The generator provides three outputs for the lighting: scaling of black parts *s*_*b*_, scaling of white parts *s*_*w*_ and a shift *t*. All outputs have the same dimensions as the image *x*. An important invariant is that the black bits of the tag must stay darker than the white bits. Otherwise, a bit could flip, and the label would change. By restricting the scaling *s*_*w*_ and *s*_*b*_ to be between 0.10 and 1, we ensure that this invariant holds. The lighting is locally corrolated and should cause smooth changes in the image. Hence, Gaussian blur *b*(*x*) is applied to *s*_*b*_, *s*_*w*_, and *t*.

(2) $$\begin{array}{r} \phi_{lighting}\left(x,s_{w},s_{b},t\right)=x\cdot b\left(s_{w}\right)\cdot W\left(x\right)+x\cdot b\left(s_{b}\right)\cdot \left(1-W\left(x\right)\right)+b\left(t\right) \end{array}$$

The segmentation mask *W*(*x*) is one for white parts and zero for the black part of the image. As the intensity of the input is distributed around −1 and 1, we can use thresholding to differentiate between black and white parts.

#### 3.2.3. Background

The background augmentation can change the background pixels arbitrarily. A segmentation mask *B*_*x*_ marks the background pixels of the image *x* which are replaced by the pixels from the generated image *d*.

(3) $$\begin{array}{r} \phi_{bg}\left(x,d\right)=x\cdot \left(1-B_{x}\right)+d\cdot B_{x} \end{array}$$

The 3D model provides the segmentation mask. As ϕ_*bg*_ can only change background pixels, the labels remain unchanged.

#### 3.2.4. Details

In this stage, the generator can add small details to the whole image including the tag. The output of the generator *d* is passed through a high-pass filter to ensure that the added details are small enough not to flip a bit. Furthermore, *d* is restricted to be in [−2, 2] to make sure the generator cannot avoid the highpass filter by producing huge values. With the range [−2, 2], the generator has the possibility to change black pixels to white, which is needed to model spotlights.

(4) $$\begin{array}{r} \phi_{detail}\left(x,d\right)=x+\text{highpass}\left(d\right) \end{array}$$

The high-pass is implemented by taking the difference between the image and a blurred version of the image (σ = 3.5). As the spotlights on the tags are only a little smaller than the bits, we increase its slope after the cutoff frequency by repeating the high-pass filter three times.

The image augmentations are applied in the order as listed above: ϕ_*detail*_ ◦ ϕ_*background*_ ◦ ϕ_*lighting*_ ◦ ϕ_*blur*_. Please note that there exist parameters to the augmentations that could change the labels. As long as it is guaranteed that such augmentations will result in unrealistic looking images, the generator network will learn to avoid them. For example, even though the detail augmentation could be used to add high-frequency noise to obscure the tag, this artifact would be detected by the discriminator.

### 3.3. Technical details

#### 3.3.1. Architecture of the generator

The generator network has to produce outputs for each augmentation. Here, we outline the most important parts. In Appendix E (Supplementary Material), we show how the network modules are connected and list all layers. The generator starts with a small network consisting of dense layers, which predicts the parameters for the 3D model (position, orientations). The output of another dense layer is reshaped and used as starting block for a chain of convolution and upsampling layers. We found it advantageous to merge a depth map of the 3D model into the generator as especially the lighting depends on the orientation of the tag in space. The input to the blur augmentation is predicted by reducing an intermediate convolutional feature map to a single scalar. An additional network is branched off to predict the input to the lighting augmentation. For the background generation, the output of the lighting network is merged back into the main generator network together with the actual image from the 3D model.

For the discriminator architecture, we mostly rely on the architecture given by Radford et al. (2015), but doubled the number of convolutional layers and added a final dense layer. This change improved the quality of the generated images.

#### 3.3.2. Clip layer

Some of the augmentation parameters have to be restricted to a range of values to ensure that the labels remain valid. The training did not converge when using functions like tanh or sigmoid due to vanishing gradients. We are using a combination of clipping and activity regularization to keep the output in a given interval [*a, b*]. If the input *x* is out of bounds, it is clipped and a regularization loss *r* depending on the distance between *x* and the appropriate bound is added.

(5) $$r(x)\;=\{\begin{array}{ll} \gamma ||x-a|{|}_{1}\; & \text{if }x<a\\ 0\; & \text{if }a\leq x\leq b\\ \gamma ||x-b|{|}_{1}\; & \text{if }x>b \end{array}$$

(6) $$\begin{array}{rc} f\left(x\right) & =\min\left(\max\left(a,x\right),b\right) \end{array}$$

With the scalar γ, the weight of the loss can be adapted. For us γ = 15 worked well. If γ is chosen too small, the regularization loss might not be big enough to move the output of the previous layer toward the interval [*a, b*]. During training, we observe that the loss decreases to a small value but never vanishes.

#### 3.3.3. Training

**Figure 5:**
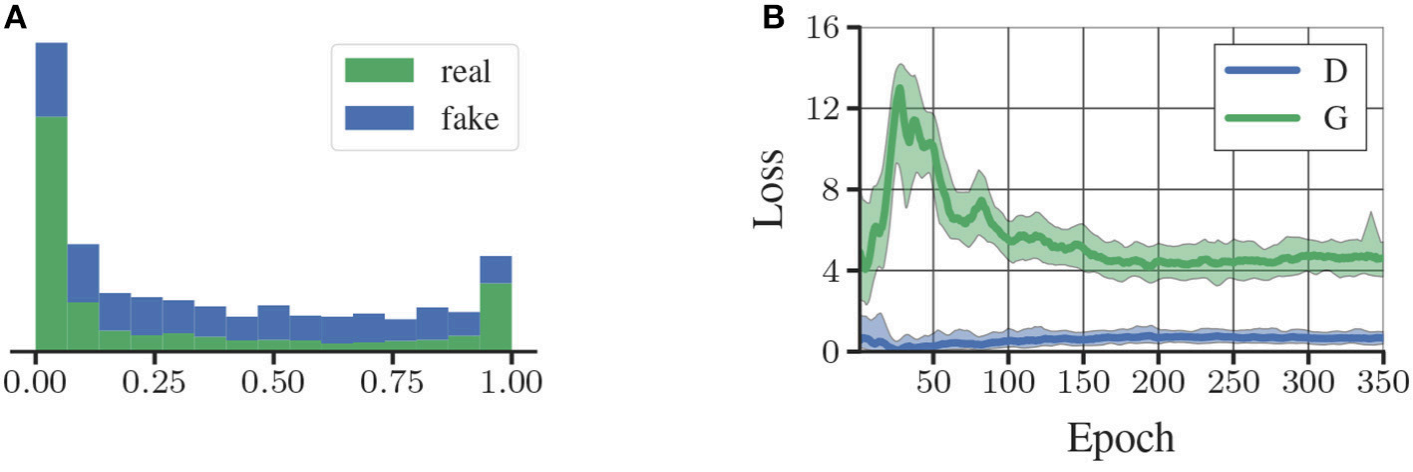
**(A)** Histogram of the discriminator scores on fake and real samples. **(B)** Losses of the generator (G) and discriminator (D).

**Figure 6:**
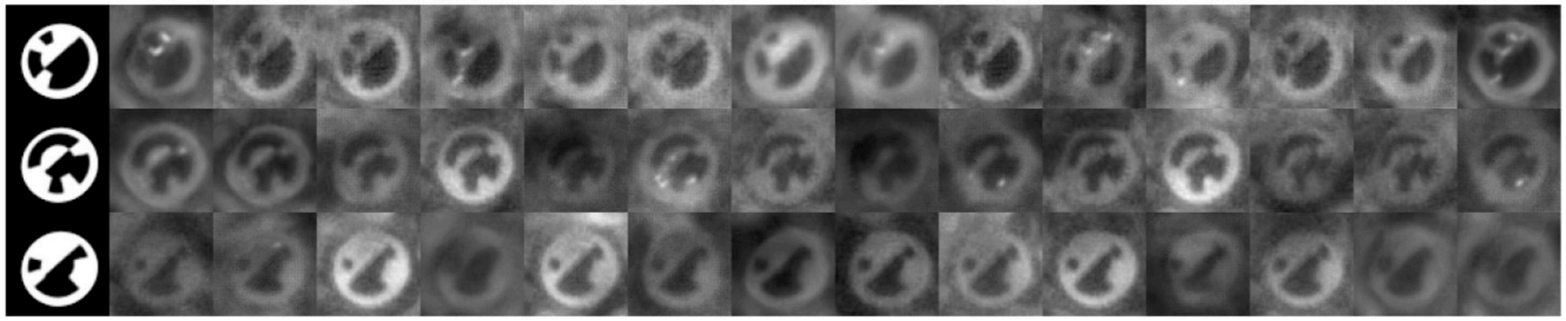
Random points in the z-space given the tag parameters.

We train generator and discriminator as in the normal GAN setting. We use 2.4 M unlabeled images of tags to train the RenderGAN. We use Adam (Kingma and Ba, 2014) as an optimizer with a starting learning rate of 0.0002, which we reduce in epoch 200, 250, and 300 by a factor of 0.25. In Figure 5B the training loss of the GAN is shown. The GAN does not converge to the point where the discriminator can no longer separate generated from real samples. There is also a difference between how real and fake tags are scored by the discriminator after training (see Figure 5A). The augmentation might restrict the generator too much such that it cannot model certain properties. Nevertheless, it is hard for a human to distinguish the generated from real images. In some cases, the generator creates unrealistic high-frequencies artifacts. The discriminator unfailingly assigns a low score to theses images. We can therefore discard them for the training of the supervised algorithm. More generated images are shown in Appendix A (Supplementary Material). In Figure 6, we show random points in the latent space, while fixing the tag parameters. The generator indeed learned to model the various lighting conditions, noise intensities, and backgrounds.

## 4. Results

We constructed the RenderGAN to generate labeled data. But does a DCNN trained with the RenderGAN data perform better than one trained on the limited amounts of real data? And are learned augmentations indeed needed or do simple hand-designed augmentation achieve the same result? The following paragraphs describe the different datasets used in the evaluation. We focus on the performance of a DCNN on the generated data. Thus, we do not compare our method to conventional GANs as those do not provide labels and are generally hard to evaluate.

### 4.1. Data from the *RenderGAN*

We generate 5 million tags with the RenderGAN framework. Due to the abundance, one training sample is only used twice during training. It is not further augmented.

### 4.2. *Real* data

The labels of the real data are extracted from ground truth data that was originally collected to evaluate bee trajectories. This ground truth data contains the path and id of each bee over multiple consecutive frames. Data from five different time spans was annotated—in total 66 K tags. As the data is correlated in time (same ids, similar lighting conditions), we assign the data from one time span completely to either the train or test set. The data from three time spans forms the train set (40 K). The test set (26 K) contains data from the remaining two time spans. The ground truth data lacks the orientation of the tags, which is therefore omitted for this evaluation. Due to the smaller size of the real training set, we augment it with random translation, rotation, shear transformation, histogram scaling, and noise (see Appendix C in Supplementary Material for exact parameters).

### 4.3. *RenderGAN + Real*

We also train a DCNN on generated and real data which is mixed at a 50:50 ratio.

### 4.4. Handmade augmentations

We tried to emulate the augmentations learned by the RenderGAN by hand. For example, we generate the background by an image pyramid where the pixel intensities are drawn randomly. We model all effects, i.e., blur, lighting, background, noise, and spotlights (see Appendix B in Supplementary Material for details on their implementation). When sampling the 5 million images from the RenderGAN, we also save the simple tags and all between steps which are then the input to the augmentations. The augmentations are applied on the fly during training. Therefore, we can rule out that the amount of training samples is responsible for the performance differences. We apply the handmade augmentation to different learned representations of the RenderGAN, e.g., we use the learned lighting representation and add the remaining effects such as background and noise with handmade augmentations (*HM LI*). See Table 1 for the different combinations of learned representations and hand designed augmentations.

**Table 1:** Datasets created with learned representations and hand-designed augmentations.

**Name**	**Learned**	**Hand-Designed**
HM 3D	3D model	Blur, lighting, background, noise, spotlights
HM LI	3D model, blur, lighting	Background, noise, spotlights
HM BG	3D model, blur, lighting, background	Noise, spotlights

### 4.5. Computer vision pipeline *CV*

The previously used computer vision pipeline (Wario et al., 2015) is based on manual feature extraction. For example, a modified Hough transformation to find ellipses. The MHD obtained by this model is only a rough estimate given that the computer vision pipeline had to be evaluated and fine-tuned on the same data set due to label scarcity.

### 4.6. Training setup

An epoch consists of 1,000 batches á 128 samples. We use early stopping to select the best parameters of the networks. For the training with generated data, we use the real training set as the validation set. When training on real data, the test set is also used for validation. We could alternatively reduce the real training set further and form an extra validation set, but this would harm the performance of the DCNN trained on the real data. We use the 34-layer ResNet architecture (He et al., 2015) but start with 16 feature maps instead of 64. The DCNNs are evaluated on the mean Hamming distance (MHD) i.e., the expected value of bits decoded wrong. Human experts can decode tags with a MHD of around 0.23.

### 4.7. Results

**Figure 7:**
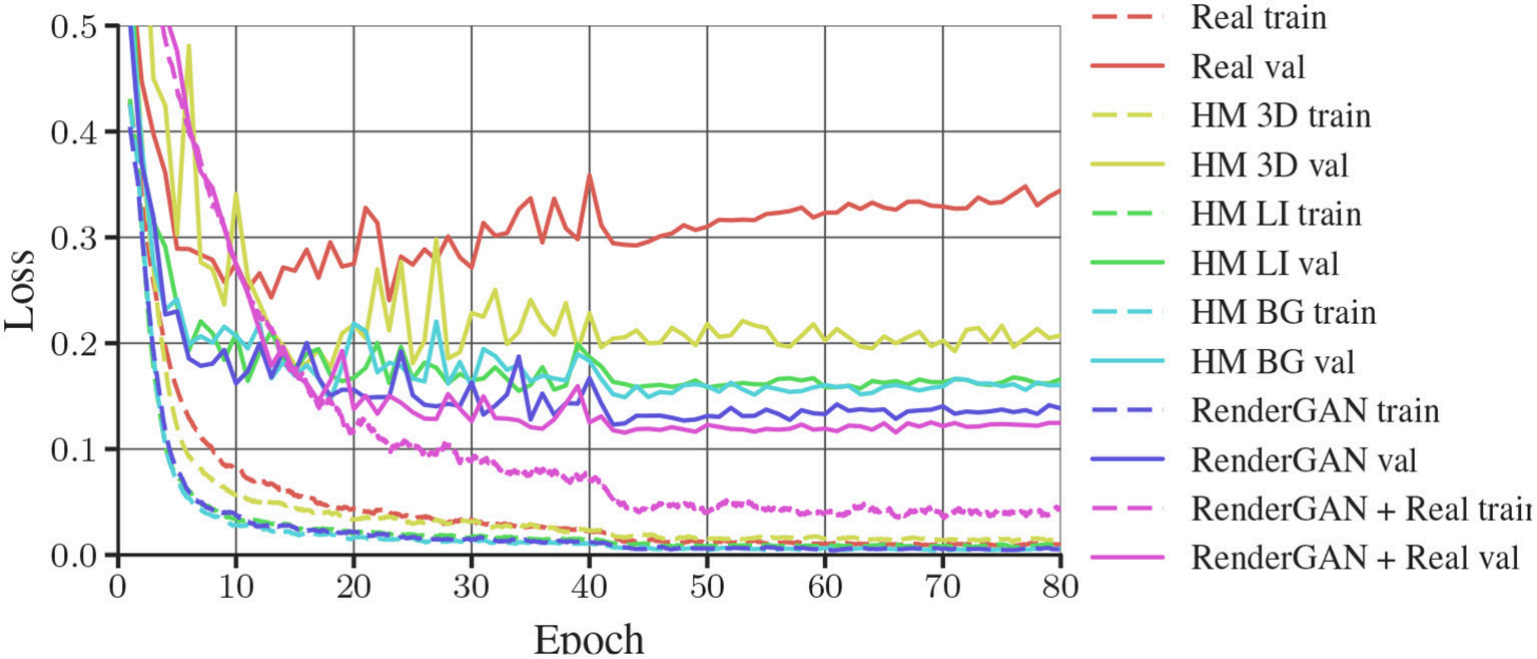
Training and validation losses of DCNNs trained on different data sets. As some data sets are missing the orientation of the tags, only the loss of the bits are plotted. Binary crossentropy is used as loss for the bits. The train and validation loss of each dataset have the same color.

In Table 2, we present the results of the evaluation. The training losses of the networks are plotted in Figure 7. The model trained with the data generated by the RenderGAN has an MHD of 0.424. The performance can furthermore be slightly improved by combining the generated with real data. The small gap in performance when adding real data is a further indicator of the quality of the generated samples.

**Table 2:** Comparison of the mean Hamming distance (MHD) on the different data sets. More samples of the training data can be found in Appendix D (Supplementary Material).

**Data**	**MHD**	**Training data**
Real	0.956	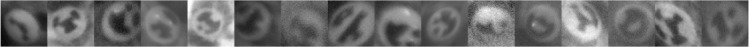
HM 3D	0.820	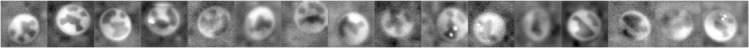
HM LI	0.491	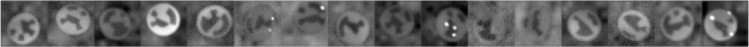
HM BG	0.505	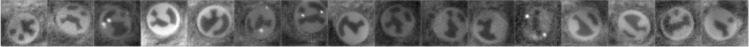
RenderGAN	0.424	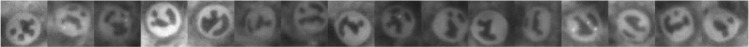
RenderGAN + Real	0.416	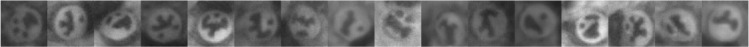
CV	1.08	

If we use predictions from this DCNN instead of the computer vision pipeline, the accuracy of the tracking improves from 55% of the ids assigned correctly to 96%. At this quality, it is possible to analyze the social behavior of the honeybees reliably.

Compared to the handmade augmentations (*HM 3D*), data from the RenderGAN leads to considerably better performance. The large gap in performance between the HM 3D and HM LI data highlights the importance of the learned lighting augmentation.

## 5. Discussion

We proposed a novel extension to the GAN framework for adding realism to a simplistic 3D object model. Compared to computer graphics pipelines, the RenderGAN can learn complex effects from unlabeled data that would be otherwise hard to model with explicit rules. Contrary to the many variants of GANs, the generator provides explicit information about the synthesized images, which can be used as labels for supervised learning. The training of the RenderGAN requires no labels.

In our project BeesBook, RenderGAN was able to generate very realistic images of bee markers. A decoder DCNN trained on this data outperforms decoders trained on various baselines. Compared to the previously used non-neural algorithm (Wario et al., 2015) RenderGAN improved the decoding accuracy significantly. Consequently the downstream tracking process that links detections through time improved in several respects. For the BeesBook project, RenderGAN was a key enabler. Without accurate ID decodings, a much larger portion of trajectories (and behaviors that we predict from them) would be assigned incorrect IDs. We have now created the largest database of honeybee trajectories consisting of approximately 4,000 animals, followed over a nine weeks recording period.

We believe that RenderGAN might be applicable to similar problems in other domains. The specific requirements of the BeesBook project can be generalized to many popular settings. Many applications could benefit from a decoder network that extracts high level properties that are costly to label, such as the pose of cars, facial features in portrait photos, or the body posture of pedestrians. Most computer vision algorithms in these domains rely already on an object model. Applying RenderGAN would then only require an additional definition of appropriate augmentations which can model the domain specific image artifacts. Some augmentations described here, such as background and the highpass filter, are very general and could be useful in other areas. Once RenderGAN is set up, arbitrary amounts of labeled data can be acquired with no further effort.

Furthermore, the RenderGAN approach can save substantial amounts of time if the data distribution changes. For example, if we would alter the marker design in the BeesBook project to include more bits, only small adaptations to the 3D model's source code and potentially some hyperparameters of the augmentations would be sufficient. In contrast, every change in object design would require new sessions of manual labeling.

## 6. Future work

For future work, it would be interesting to see the RenderGAN framework used on other tasks, e.g., human faces, pose estimation, or viewpoint prediction. In different contexts, different augmentations may be required, for example, colorization, affine transformations, or diffeomorphism. The RenderGAN could be especially valuable to domains where pre-trained models are not available or when the annotations are complex. Another direction of future work might be to extend the RenderGAN framework to other fields. For example, in speech synthesis, one could use an existing software synthesizer as a basic model and improve the realism of the output with a similar approach as in the RenderGAN framework.

## Ethics statement

German law does not require approval of an ethics committee for studies involving insects.

## Author contributions

LS, BW, and TL: conceptualization, data curation, writing-original draft, writing-review and editing; LS: methodology, software, visualization; TL: resources, supervision, project administration.

### Conflict of interest statement

The authors declare that the research was conducted in the absence of any commercial or financial relationships that could be construed as a potential conflict of interest.
