# Supplementary material for: RenderGAN: Generating Realistic Labeled Data
Source: Front Robot AI. 2018 Jun 8;5:66. doi: 10.3389/frobt.2018.00066 (PMC7805882; doi:10.3389/frobt.2018.00066)
Supplement: Supplementary file 1 [file Data_Sheet_1.pdf]

Appendices

A Generated Images

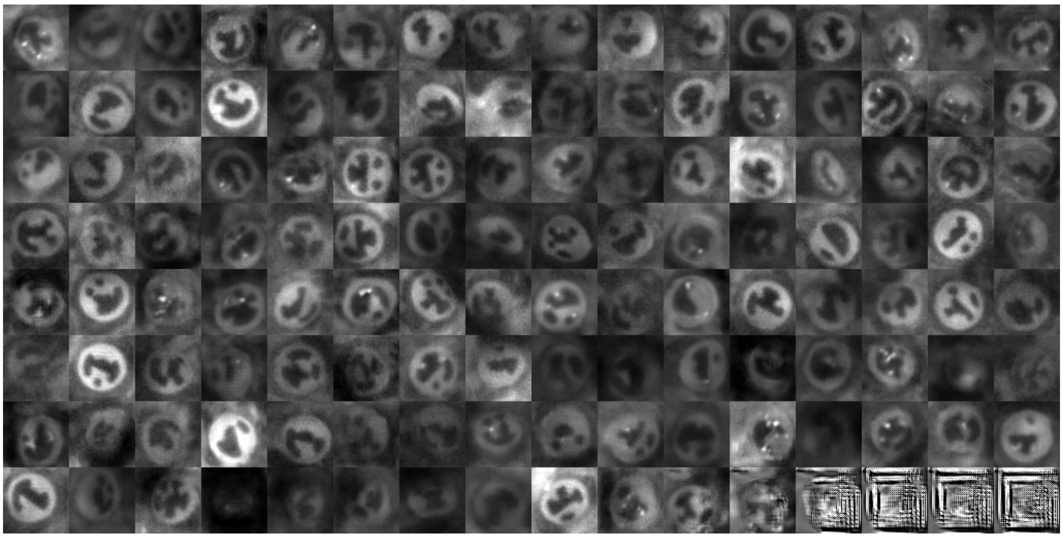

(a) Generated images

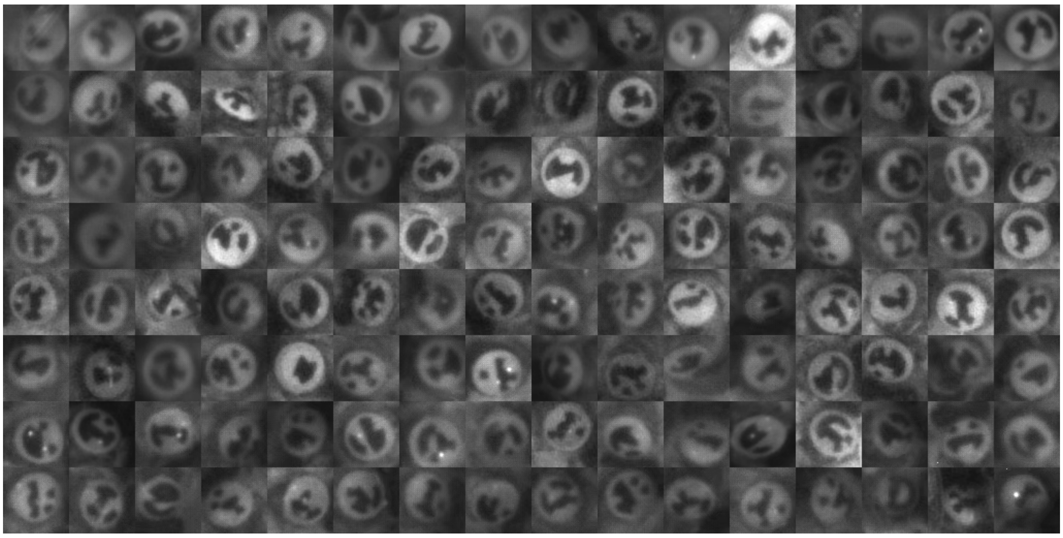

(b) Real images

Figure S1: Continuum visualization on the basis of the discriminator score: Most realistc scored samples top left corner to least realistc bottom right corner. Images with artifacts are scored unrealistic and are not used for training.

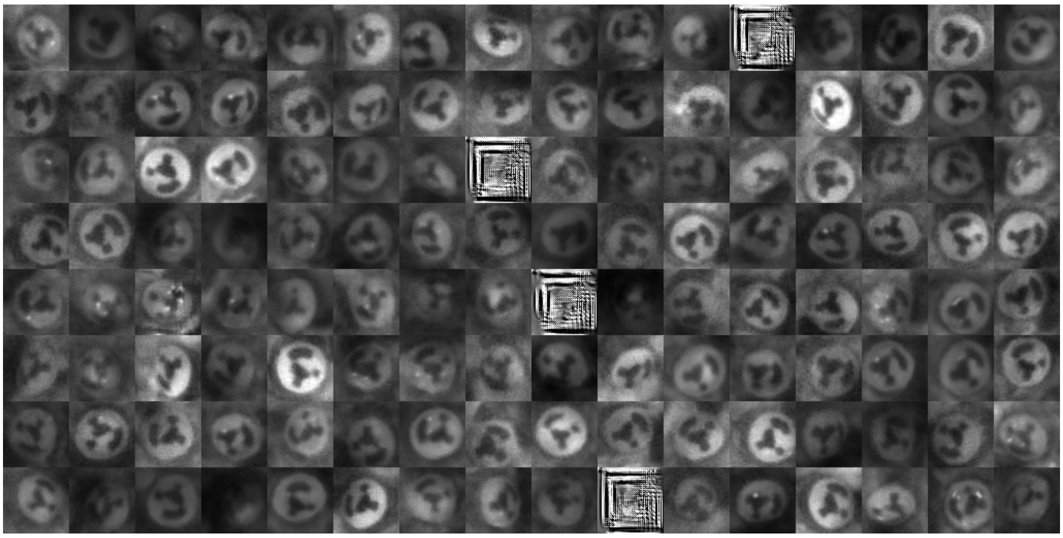

Figure S2: Images generated with the generator given a fixed bit configuration

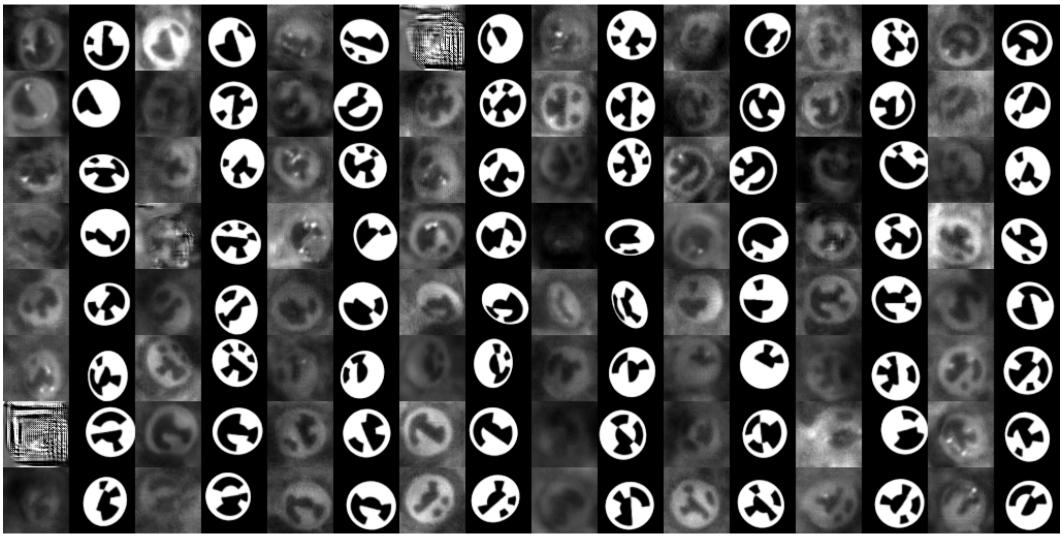

Figure S3: Correspondence of generated images and 3D model

B HANDMADE AUGMENTATIONS

We constructed augmentations for blur, lighting, background, noise and spotlights manually. For synthesizing lighting, background and noise, we use image pyramids, i.e. a set of images $L_0, \ldots, L_6$ of size $(2^i \times 2^i)$ for $0 \leq i \leq 6$. Each level L_i in the pyramid is weighted by a scalar ω_i. Each pixel of the different level L_i is drawn from $\mathcal{N}(0, 1)$. The generated image I_6 is given by:

$$I_0 = \omega_0 L_0 \tag{S1}$$

$$I_i = \omega_i L_i + \text{upscale}(I_{i-1}) \tag{S2}$$

, where upscale doubles the image dimensions. The pyramid enables us to generate random images while controlling their frequency domain by weighting the pyramid levels appropriately.

- **Blur:** Gaussian blur with randomly sampled scale.
- **Lighting:** Similar as in the RenderGAN. Here, the scaling of the white and black parts and shifting is constructed with image pyramids.
- **Background:** image pyramids with the lower levels weight more.
- **Noise:** image pyramids with only the last two layer.
- **Spotlights**: overlay with possible multiple 2D Gauss function with a random position on the tag and random covariance.

We selected all parameters manually by comparing the generated to real images. However, using slightly more unrealistic images resulted in better performance of the DCNN trained with the HM 3D data. The parameters of the handmade augmentations can be found online in our source code repository.

C AUGMENTATIONS OF THE REAL DATA

We scale and shift the pixel intensities randomly, i.e. $sI + t$, where I is the image and s, t are scalars. The noise is sampled for each pixel from $\mathcal{N}(0, \epsilon)$, where $\epsilon \sim \max(0, \mathcal{N}(\mu_n, \sigma_n))$ is drawn for each image separately. Different affine transformations (rotation, scale, translation, and shear) are used.

Table S1: Parameters of the augmentation of the real data

Name	Distribution
Intensity Scale (s)	unif(0.9, 1.1)
Intensity Shift (t)	unif(-0.2, 0.2)
Noise Mean (μ_n)	0.04
Noise Std (σ_n)	0.03
Rotation	unif(0, 2π)
Scale	unif(0.7, 1.1)
Shear	unif(-0.3, 0.3)
Translation	unif(-4, 4)

D TRAINING SAMPLES

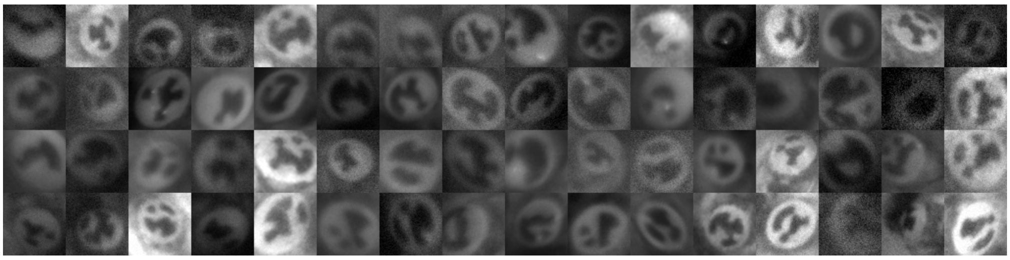

(a) Real trainings samples

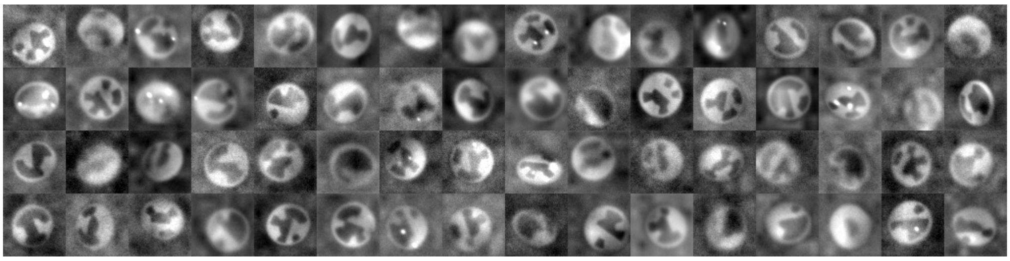

(b) HM 3D training samples

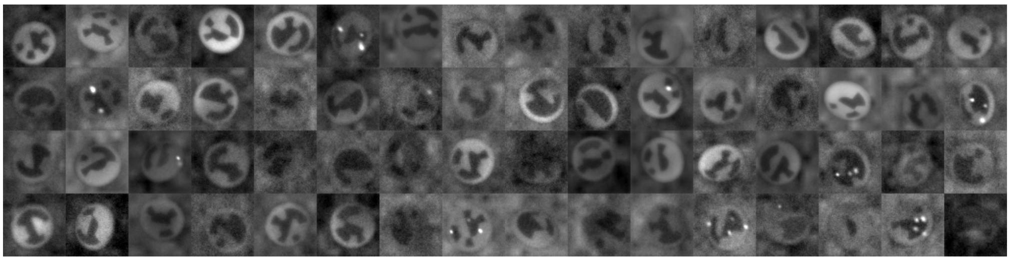

(c) HM LI training samples

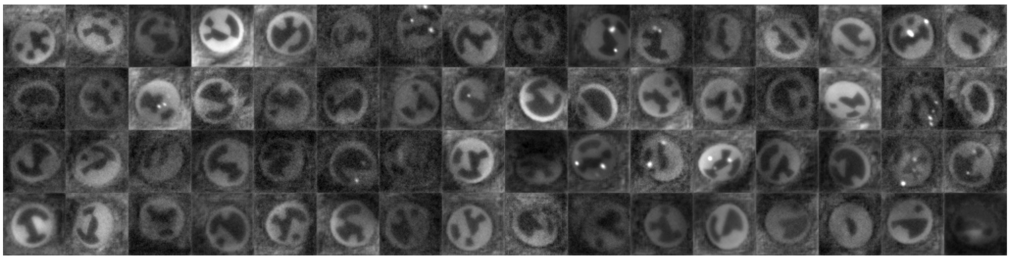

(d) HM BG training samples

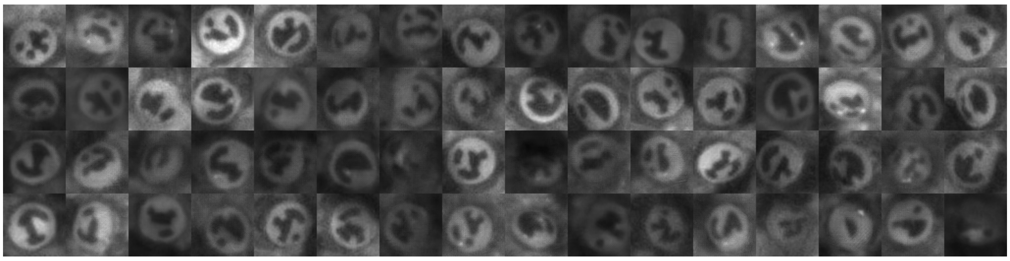

(e) RenderGAN

Figure S4: Training samples from the different datasets.

E NETWORK ARCHITECTURE

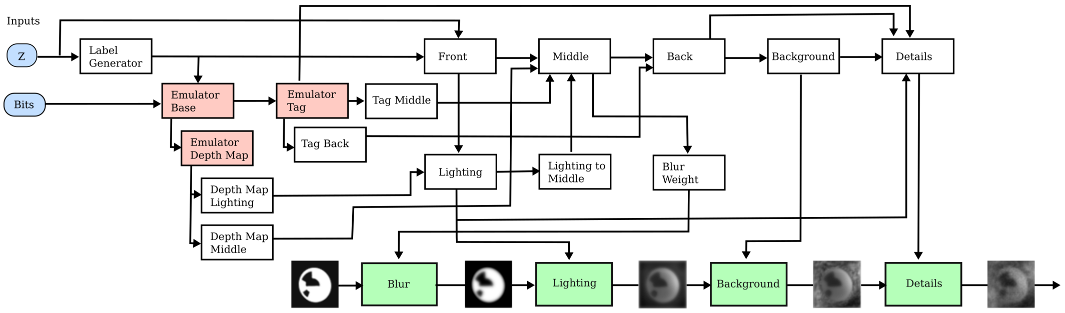

Figure S5: Topology of the generator. Each box represents a neural network module. The tag emulator network (red boxes) is trained to simulate the 3D model and is fixed during training of the generator. The augmentation functions (green boxes) receive their parameters from different network modules of the generator. The initial input is obtained from the tag emulator network.

An overview of the different network moudles is shown in Fig. S5. Below, the layers of each network module are listed. All `Conv2d` layers have a kernel size of 3x3 if not other specified. The layers are allways applied sequentially. `BN` stands for batch normalization.

```
# Inputs:
Noise Vector: Z @ (50)
Tag Bits: Bits @ (12)

# Label Generator
Input: Random noise z @ (50)
Dense(64), BN, Dropout(0.25), ReLU,
Dense(64), BN, Dropout(0.25), ReLU,
labels_without_bits = Dense(12), BN, InBounds(-1, 1)

# Emulator Base
Input: Label Generator, Bits
Dense(256), ReLU,
Dense(1024), ReLU,
Dense(2048), ReLU,
Reshape @ (128, 4, 4)
Conv2D(128), ReLU,
Conv2D(128), ReLU,
UpSampling2D() @ (128, 8, 8)
Conv2D(64), ReLU,
Conv2D(64), ReLU,
UpSampling2D() @ (64, 16, 16)
Conv2D(32), ReLU,
Conv2D(32), ReLU,

# Emulator Tag
Input: Emulator Base
Conv2D(32), ReLU,
Conv2D(32), ReLU,
UpSampling2D() @ (64, 32, 32)
Conv2D(32), ReLU,
Conv2D(32), ReLU,
UpSampling2D() @ (32, 64, 64)
Conv2D(32), ReLU,
tag = Conv2D(32, kernel_size=1) @ (1, 64, 64)

# Emulator Depth Map
Input: Emulator Base
Conv2D(16, 1),
depth_map = Conv2D(32, kernel_size=1) @ (1, 16, 16)

# Front
Input: Merge(Label Generator, Z)
Dense(8192), BN, ReLU
Reshape @ (512, 4, 4)
Upsampling @ (512, 8, 8)
Conv2D(256, 3), BN, ReLU
Conv2D(256, 3), BN, ReLU
Upsampling @ (512, 16, 16)
```

```
Conv2D(128, 3), BN, ReLU
Conv2D(128, 3), BN, ReLU

# Depth Map Lighting
Input: Emulator Depth Map @ (1, 16, 16)
Conv2D(32, 3) @ (32, 16, 16), BN, ReLU
Conv2D(32, 3), BN, ReLU

# Depth Map Middle
Input: Emulator Depth Map @ (1, 16, 16)
Conv2D(32, 3), BN, ReLU
Conv2D(32, 3), BN, ReLU

# Lighting
Input: Merge(Depth Map Lighting, Front)  @ (160, 16, 16)
Conv2D(64, 3), BN, ReLU
MaxPooling(2) @ (64, 8, 8)
Conv2D(64, 3), BN, ReLU
Conv2D(64, 3), BN, ReLU
UpSampling(2) @ (64, 16, 16)
Conv2D(64, 3), BN, ReLU
UpSampling(2) @ (64, 32, 32)
Conv2D(64, 3), BN, ReLU
Conv2D(3, 3)
UpSampling(2) @ (3, 32, 32)
GaussianBlur(sigma=2.5),
light_outs = InBounds(0, 2),

# Lighting to Middle
Conv2D(32, 3) @ (32, 64, 64)
MaxPooling(2) @ (32, 32, 32)
BN, ReLU
Conv2D(32,  3),
MaxPooling(2) @ (32, 16, 16),
BN, ReLU

# Tag Middle
Input: Tag of 3D Emulator @ (1, 64, 64)
Rescale(2) @ (1, 32, 32)
Conv2D(16, 3)
MaxPooling(2) @ (16, 16, 16)
BN, ReLU,
Conv2D(16, 3), BN, ReLU

# Tag Back
Input: Tag of 3D Emulator @ (1, 64, 64)
Rescale(2) @ (1, 32, 32)
Conv2D(16, 3), BN, ReLU,
Conv2D(16, 3), BN, ReLU,

# Middle
Merge(
    Front,
    Tag Middle,
    Lighting to Middle,
    Depth Map Middle,
)
Upsampling(2) @ (208, 32, 32)
Conv(128, 3), BN, ReLU
Conv(128, 3), BN, ReLU
Conv(128, 3), BN, ReLU

# Back
Merge(Middle, Tag Back)
Upsample(2) @ (144, 64, 64)
Conv2d(64, 3) @ (64, 64, 64), BN, ReLU
Conv2d(64, 3), BN, ReLU

# Background
Input: Back
Conv2d(1, 3),
background_generated = InBounds(-1, 1)

# Blur Weight
Input: Middle
Conv2D(1),
Flatten() @ (900)
Dense(1)
InBounds(0.25, 1.)

# Details
```

```
Merge(Background, Tag, Back, Lighting)
Conv2D(64, 3) @ (64, 64, 64), BN, ReLU
Conv2D(64, 3), BN, ReLU
Conv2D(64, 3), BN, ReLU
Conv2D(1),
high_freq = HighPass()

# Augmentations
Names are the same as used in the code.

Segmentation(Tag)
tag_blur = BlendingBlur(tag_blur, Blur Weight)
tag_lighten = AddLighting(tag_blur, light_outs)
tag_background = Background(tag_lighten, background_generated)
fake = Sum(tag_background, high_freq)

# Discriminator
All LeakyReLU layers have 0.2 as leaky coefficient.

Conv2d(32, kernel_size=5, subsample=2), LeakyReLU @ (32, 32, 32)
Conv2d(64, subsample=2), BN, LeakyReLU @ (64, 16, 16)
Conv2d(64), BN, LeakyReLU
Conv2d(128, subsample=2), BN, LeakyReLU @ (128, 8, 8)
Conv2d(128), BN, LeakyReLU
Conv2d(256, subsample=2), BN, LeakyReLU @ (256, 4, 4)
Conv2d(256), BN, LeakyReLU
Flatten() @ (4096)
Dense(512), BN, LeakyReLU
Dense(1), Sigmoid
```
